# Inhibition of CXCR4 by LY2624587, a Fully Humanized Anti-CXCR4 Antibody Induces Apoptosis of Hematologic Malignancies

**DOI:** 10.1371/journal.pone.0150585

**Published:** 2016-03-08

**Authors:** Sheng-Bin Peng, Xiaoyi Zhang, Donald Paul, Lisa M. Kays, Ming Ye, Peter Vaillancourt, Michele Dowless, Louis F. Stancato, Julie Stewart, Mark T. Uhlik, Haiyan Long, Shaoyou Chu, Victor H. Obungu

**Affiliations:** Lilly Research Laboratories, Lilly Corporate Center, Indianapolis, Indiana, 46285, United States of America; University of Kansas Medical Center, UNITED STATES

## Abstract

SDF-1 and CXCR4 are a chemokine and chemokine receptor pair playing critical roles in tumorigenesis. Overexpression of CXCR4 is a hallmark of many hematological malignancies including acute myeloid leukemia, chronic lymphocytic leukemia and non-Hodgkin’s lymphoma, and generally correlates with a poor prognosis. In this study, we developed a humanized anti-CXCR4 monoclonal antibody, LY2624587 as a potent CXCR4 antagonist that was advanced into clinical study for cancer. LY2624587 blocked SDF-1 binding to CXCR4 with an IC_50_ of 0.26 nM, and inhibited SDF-1-induced GTP binding with a K_b_ of 0.66 nM. In human lymphoma U937 and leukemia CCRF-CEM cells expressing endogenous CXCR4, LY2624587 inhibited SDF-1-induced cell migration with IC_50_ values of 3.7 and 0.26 nM, respectively. This antibody also inhibited CXCR4 and SDF-1 mediated cell signaling including activation of MAPK and AKT in tumor cells expressing CXCR4. Bifocal microscopic and flow cytometry analyses revealed that LY2624587 mediated receptor internalization and caused CXCR4 down-regulation on the cell surface. In human hematologic cancer cells, LY2624587 caused dose dependent apoptosis *in vitro* and *in vivo*. In mouse xenograft models developed with human leukemia and lymphoma cells expressing high levels of CXCR4, LY2624587 exhibited dose-dependent tumor growth inhibition and provided significant survival benefit in a disseminated lymphoma model. Collectively, we have demonstrated that CXCR4 inhibition by LY2624587 has the potential for the treatment of human hematological malignancies.

## Introduction

A large body of scientific literature has reported that Stromal cell Derived Factor 1 (SDF-1, or CXCL12) and CXCR4, a chemokine and chemokine receptor pair play important roles in multiple phases of tumorigenesis, including tumor cell proliferation, survival, invasion and metastasis, and angiogenesis [1–5]. Preclinical data have validated CXCR4 as a potential target for anti-cancer drug development. It has been demonstrated that CXCR4 is constitutively expressed in a variety of human cancers, such as breast, kidney, lung, colon, and brain cancer [1–10]. Several forms of hematologic malignancies including leukemia and lymphoma are also shown to express high levels of CXCR4 [11, 12]. The expression is especially noticeable in advanced and metastatic cancers and generally linked to cancer pathogenesis and outcome. Additionally, SDF-1, the only known ligand for CXCR4, is a well characterized survival and angiogenic factor [13–15]. It is highly expressed in the tumor microenvironment, bone marrow, lung, liver, and lymph nodes, the organ sites most commonly involved in tumor metastasis [3, 4]. The interaction of SDF-1 and CXCR4 activates multiple signal transduction pathways in tumor cells, including PI3K/AKT and MAPK cascade, which are the two important pathways involved in cell survival and proliferation [16–18]. Therefore, the CXCR4 and SDF-1 axis offers a therapeutic opportunity for anti-cancer drug development.

In recent years, significant effort has been invested in the development of small molecule and peptide inhibitors in interrupting the CXCR4 and SDF-1 axis for anti-HIV infection, cancer and other human diseases [19, 20]. However, it is quite challenging to develop an agent suitable for clinical use due to the nature of the SDF-1 and CXCR4 interaction. SDF-1 is a highly basic and cationic chemokine, and contains approximately 25% basic residues [13]. For development of a small molecule inhibitor against this interaction, multiple basic centers in a molecule are generally required to achieve high affinity in blocking of the SDF-1 and CXCR4 interaction [19]. One such a small molecule inhibitor is the well characterized bicyclam AMD3100 [21]. Due to its basic property, AMD3100 is not orally bioavailable, causes significant safety concerns in a chronic dose schedule due to compound-associated toxicity [22], and can only be used for a short time treatment in humans. Alternatively, many potent peptide antagonists of CXCR4 have been identified, but these peptide antagonists generally have very short in vivo half-lives and poor pharmacokinetic (PK) properties [20]. Two such peptide antagonists are BTK140 [23] and CTCE-9908 [24], both of which have been advanced into clinical studies.

In this study, we describe the development of a fully humanized anti-CXCR4 monoclonal antibody, LY2624587 as a potent and selective CXCR4 antagonist that entered into clinical studies (NCT01139788). LY2624587, along with MDX-1338 [25], are among the first in class anti-CXCR4 monoclonal antibody developed for cancer. It is potent in blocking SDF-1 binding to CXCR4, induces receptor internalization and inhibits CXCR4 and SDF-1 mediated downstream signaling. In human hematologic cancer cells, LY2624587 induced dose dependent apoptosis in vitro and in vivo. In tumor xenograft models developed with human leukemia and lymphoma cells, this antibody exhibited dose-dependent tumor growth inhibition, and displayed a statistically significant survival benefit in a disseminated liquid model developed with human lymphoma cells. Therefore, inhibition of CXCR4 by LY2624587 may represent a potential treatment option for patients with hematological malignancies.

## Materials and Methods

### Reagents

Anti-ERK1/2, anti-phospho-ERK1/2 and anti-phospho-Akt polyclonal antibodies were purchased from Cell Signaling (Beverly, MA). Anti-β-actin monoclonal antibody was from Sigma (St Louis, MO). Anti-hCXCR4 monoclonal antibody for immuno-fluorescent staining was from R&D Systems (Minneapolis, MN). Recombinant human SDF-1α was purchased from PeproTech EC Ltd. (London, UK). Alexafluor- 488 dye was obtained from Molecular Probes. LY2624587 is a recombinant antibody and available through a material transfer agreement with Eli Lilly and Company.

### Cell culture

Human lymphoblastic leukemia CCRF-CEM cells, non-Hodgkin’s lymphoma Namalwa cells, and histiocytic lymphoma U937 cells were purchased from the American Type Culture Collection (Manassas, VA) through 2004–2009. All these cells were passaged for fewer than 2 months after which time new cultures were initiated from vials of frozen cells. Characterization of the cell lines was done by a third party vendor (RADIL, Columbia, MO), which included profiling (by PCR) for contamination by various microorganisms of bacterial and viral origin, no contamination was detected. The samples were also verified to be of human origin without mammalian inter-species contamination. The alleles for 9 different genetic markers were used to determine that the banked cells matched the genetic profile that has been previously reported. Cells were grown in RPMI 1640 or DMEM medium supplemented with 10% (v/v) FBS. Cell cultures were passaged twice each week, and maintained in an incubator at 37°C in an atmosphere of 5% CO_2_ and 95% air.

### [^125^I] SDF-1α/human CXCR4 binding assay

CCRF-CEM cells expressing endogenous CXCR4 and ^125^I-labeled SDF-1α were used in a binding assay as described [18]. Typically, the assay was performed in a 96-well U-bottom, non-treated polystyrene plate (Corning Incorporated). The binding assay buffer was prepared with RPMI 1640 medium (Gibco) containing 10 mM HEPES, pH 7.5, and 0.2% BSA. Briefly, 200 μL reaction mixtures containing 300 pM ligand, 60 pM [^125^I] SDF-1α (Perkin Elmer) plus 240 pM cold SDF-1 (R&D Systems), different concentrations of LY2624587, 100,000 CEM cells, and 0.5 mg SPA beads (wheatgerm agglutinin beads; Amersham) were incubated at room temperature for 2 hours. Bound ^125^I-SDF-1α was detected using a 1450 Microbeta Liquid Scintillation and Luminescence Counter (Wallac) in SPA mode.

### GTP binding assay

GTP-γ^35^S binding was measured in a 96 well format using a modified antibody capture technique previously described [18, 26]. Antagonist dose responses were performed in the presence of an 80% efficacious dose of full agonist (SDF-1α) in GTP-binding assay buffer (20 mM Hepes, 100 mM NaCl, 1 mM MgCl_2_, 1 μM GDP, pH 7.4). CEM membrane prepared by sucrose density gradient centrifugation, SDF-1α, and compounds were combined and incubated for 15 minutes at room temperature. 500 pM GTP-γ-^35^S (PerkinElmer) was added with a subsequent 35 minute incubation. This was followed by the addition of 0.27% Nonidet P40 detergent (Roche) and anti-Gi antibody (final dilution of 1:400; Covance) each followed by a 15 minute incubation. 1.25 mg of anti-rabbit antibody scintillation proximity assay bead (GE Healthcare) was then added, and plates were incubated for an additional hour. Plates were centrifuged at 700x*g* for 10 minutes and bound GTP-γ-^35^S was detected using a Wallac MicroBeta TriLux scintillation counter (PerkinElmer).

### Cell migration (chemotaxis) assay

A cell migration assay was developed using U937 and CCRF-CEM cells expressing endogenous CXCR4 as described [18]. Briefly, U937 or CCRF-CEM cells were harvested and washed once with chemotaxis assay buffer prepared with 1x RPMI medium containing 10 mM HEPES, pH 7.5, and 0.3% BSA. Cells were then resuspended in assay buffer at a density of 5x10^6^ cells/mL. The assay was performed in a 96-well ChemoTx plate (NeuroProbe). Generally, 50 μL of cell mixture with or without LY2624587 was plated on the upper chamber, and 30 μL of SDF-1α (10 ng/mL) prepared in 1x chemotaxis buffer was added to the lower chamber. The plate was then incubated for 2.5 hours at 37°C. Following the incubation, 5 μL of CellTiter 96 AQ (Promega) were added into the lower chamber. The plate was then incubated for 60 minutes at 37°C, and the migrated cells were detected by measuring the absorbance at 492 nm with a Tecan Spectrafluor Plus Microplate Reader (Salzburg, Austria).

### Western blot analysis

The treatment of CCRF-CEM and Namalwa cells with SDF-1, cell lysate preparation and Western blot analysis were performed as described previously [17].

### Antibody-mediated receptor internalization

To demonstrate if LY2624587 induced receptor-mediated internalization, LY2624587 was labeled with fluorescent dye Alexa 488 as described by the manufacturer. The labeled antibody was then used to treat MDA-MB-435/CXCR4 stably transfected cells. Briefly, 1x10^5^ MDA-MB-435/CXCR4 cells were seeded in the glass bottom of culture dishes (MatTek, part No. P35GC-1.0-14-C) and cultured overnight. The cells were then incubated with 4μg/mL of LY2624587 for 1 or 2 hours at 37°C. In one condition, cells were incubated with labeled LY2624587 first, then fixed with 2% formaldehyde for 10 min. In another condition, the cells were fixed with 2% formaldehyde for 10 min first, then incubated with Alexa 488-labeled LY2624587. After these treatments, the cells were examined with the Zeiss LSM510 confocal microscope using 488 nm laser excitation to collect 505 nm-530 nm emission with the 40x C-Apo 40x/NA 1.2 water immersion objective for localization of receptor-antibody complex.

### Annexin V staining and analysis by flow cytometry

Briefly, Namalwa or ARH-77 cells in growth medium containing 1% FBS were treated with different concentrations of LY2624587 for 48 hours, then stained with anti-annexin V antibody conjugated with FITC (R&D Systems). After a brief PBS wash, the cells were re-suspended in PBS buffer for flow cytometry analysis in Beckman Coulter FC 500 Cytomics flow cytometry.

### Caspase 3 and nuclear fragmentation detection by Cellomics

Namalwa or CCRF-CEM cells were treated with different concentrations of LY2624587 for 2 to 4 days in growth medium containing 10% FBS. After treatment, cells were fixed with 3.7% formaldehyde and washed in PBS. Cells were permeabilized with 0.1% Triton X-100 in PBS, washed and blocked in PBS containing 1% BSA. Cells were then incubated for 1 hour with rabbit anti-activated Caspase3 polyclonal antibody (BD Biosciences) diluted in PBS with 1% BSA. Cells were washed 2 times with PBS then incubated for 1 hour with Alexa Fluor 488 goat anti Rabbit IgG (Invitrogen) and 200 ng/mL Hoechst 33342 (Invitrogen) diluted in PBS with 1% BSA. Stained plates were scanned using ArrayScan Vti (Thermo Fisher) and the Target Activation bioapplication was used for quantitation of fluorescent signal.

### *In vivo* studies

The Eli Lilly and Company Animal Care and Use Committee approved all the in vivo protocols and protocol changes that included study termination due to some tumors with exceeding tumor size limits. For the non-Hodgkin’s lymphoma Namalwa model, 200,000 Namalwa cells mixed with matrigel (1:1) were implanted subcutaneously into the rear flank of SCID female mice from Jackson Laboratories. For the acute lymphoblastic leukemia CCRF-CEM model, 5x10^6^ cells mixed with matrigel (1:1) were implanted subcutaneously into the rear flank of SCID female mice. In both models, the implanted tumor cells grew as solid tumors. To test the efficacy of LY2624587 in these models, the animals (9 or 10 each group) were treated with 1 μg/mouse (0.04 mg/kg), 10 μg/mouse (0.4 mg/kg), 30 μg/mouse (1.2 mg/kg), or 100 μg/mouse (4 mg/kg) of LY2624587. The antibody was subcutaneously administered once every 4 days and 7 days post tumor cell implant, and the tumor volume and body weight were measured every 2 or 3 days. Anti-tumor growth activity was determined by the reduction in tumor volume in treatment groups compared to the tumor volume in control groups treated with vehicle alone or isotype IgG. To elucidate the molecular mechanism of anti-tumor activity, a high-content multiplexed tissue imaging and quantification method was utilized for tumor tissue staining of nuclear intensity, TUNEL area, CD31 and Ki67 as described (27). For quantification of TUNEL area, eight entire 4 micron slides from a whole tumor were scanned and counted. The percentage of total apoptosis area = 100xTUNEL Phantom count/total Hoechst Phantom count [27].

For the disseminated liquid lymphoma model, 200,000 Namalwa cells were intravenously injected into SCID mice via tail vein. To determine the anti-tumor growth activity of LY2624587 in this model, animals were treated with 30 or 100 μg/mouse (i.e. 1.2 or 4 mg/kg) of LY2624587, or control IgG 24 hours post tumor cell injection. The antibody was dosed subcutaneously once every four days for 6 weeks, and the animal mortality was monitored on daily basis. The survival probability over time was plotted in a Kaplan-Meier survival curve. For this survival study, animals were euthanized when any signs of pain or distress were observed such as labored breathing, lethargy, lack of movement, hunched posture, paralysis, >20% body weight loss, unable to reach food or water, etc. Animals were generally observed twice daily, and veterinary staff was called immediately if adverse events were observed prior to onset of morbidity. Animals were euthanized by CO2 inhalation followed by cervical dislocation if needed. There were 14 animals that died in this study and 53 that were euthanized due to signs of pain or distress (specifically lethargy, hindlimb paralysis, thin, hunched posture). No analgesics were used, and animals were immediately euthanized when distress was noted.

### Human patient samples and flow cytometry analysis

The blood samples of human chronic lymphocytic leukemia (CLL) patients were freshly collected from Methodist Hospital, Indiana University at Indianapolis. Clarian Health Review Board of Ethics Committee specifically approved this study. Generally 20 ml of fresh blood sample from each patient was collected in heparin tube, stored on ice, and subjected to FACS analysis in Beckman Coulter FC 500 Cytomics flow cytometry with a PE-conjugated CXCR4 antibody (R&D Systems) and a FITC conjugated CD19 antibody (R&D Systems). After acquiring data, the histograms were analyzed with ModFit LT 3.0 (Verity House Software).

## Results

### LY2624587 is a potent and inhibitory anti-CXCR4 monoclonal antibody

**Fig 1:**
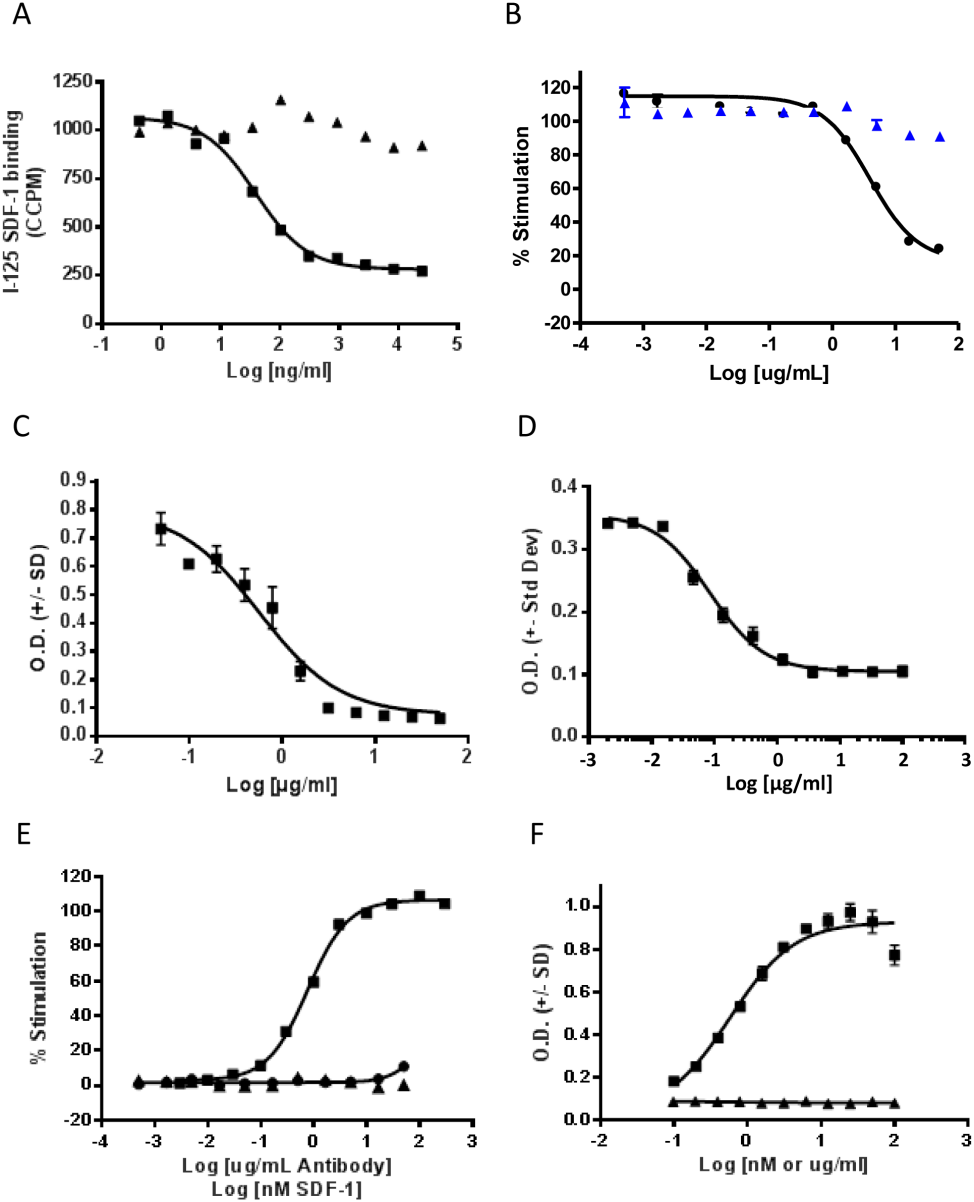
*In vitro* activities of anti-CXCR4 monoclonal antibody LY2624587. A. Inhibition of [^125^I] SDF-1α binding to CXCR4 by LY2624587 (square) or control IgG (triangle). Ligand binding assay was developed with CCRF-CEM cells, and the CCPM in the Y axis stands for corrected counts per minute. B. Dose dependent inhibition of SDF-1 induced GTP binding by LY2624587 (solid circle) or control IgG (triangle). GTP binding assay was developed with CCRF-CEM membrane. C. Inhibition of SDF-1 induced cell migration (chemotaxis) in U937 cells by LY2624587. D. Inhibition of SDF-1 induced cell migration (chemotaxis) in CCRF-CEM cells by LY2624587. E and F. LY2624587 has no apparent agonist activity. LY2624587 was tested in GTPγS35 binding assay (E) or cell migration assay (F) in agonist mode. The GTPγS35 binding assay was developed with CCRF-CEM cell membrane, and the SDF-1-induced cell migration assay was developed with U937 cells as described under “Materials and Methods.”

LY2624587 was derived from a low affinity mouse anti-human CXCR4 antibody which had been generated by immunizing mice with Chinese Hamster Ovary (CHO) cell line expressing human CXCR4. This mouse antibody was humanized and affinity matured to result in a high affinity humanized antibody, LY2624587. The humanized antibody was developed as a human IgG4 with a serine to proline mutation in the hinge region to eliminate half antibody phenomenon associated with human IgG4 antibody isotypes. Additional optimization included further engineering in the Fc region to reduce antibody effector functions and elimination of potential glycosylation sites in the CDR regions. As shown in Fig 1A, LY2624587 inhibited SDF-1α binding to human CXCR4 in a dose-dependent manner with an IC_50_ of 0.259 nM, suggesting that LY2624587 interacts with human CXCR4 with high affinity and consequently blocks SDF-1 binding. To further confirm the inhibitory activity of LY2624587, an SDF-1-induced GTP binding assay was developed with radioactively labeled GTPγS35 and purified CEM cell membranes. As demonstrated in Fig 1B, LY2624587 inhibited SDF-1-mediated GTPγS35 binding in a dose-dependent manner with a Kb of 0.66 nM. This observation suggests that LY2510924 functions as an antagonist of CXCR4. One of the important functions of the CXCR4/SDF-1 interaction is to regulate cell migration. To determine the cellular activity of LY2624587, we developed cell migration (or chemotaxis) assays with U937 and CCRF-CEM cells, both of which express endogenous CXCR4. As demonstrated in Fig 1C and 1D, LY2624587 inhibited SDF-1-induced migration of U937 or CCRF-CEM cells in a concentration dependent manner with an IC_50_ of 3.7 and 0.26 nM (562 and 85 ng/mL), respectively, further confirming that LY2624587 acted as a CXCR4 inhibitor.

### LY2624587 has no apparent agonist activity

Functional studies from cell migration, GTP binding and cell signaling analysis (see results below) demonstrated that LY2624587 inhibited SDF-1/CXCR4-mediated cellular functions, suggesting that it acted as an antagonist of CXCR4. Since LY2624587 induced receptor internalization, we tested it, in an agonist mode, in GTPγS35 binding and cell migration assays to determine if LY2624587 had any agonist activity. As illustrated in Fig 1E and 1F, SDF-1α, the natural ligand of CXCR4, stimulated GTPγS35 binding and cell migration in a concentration-dependent manner. However, across a wide range of concentrations tested for LY2624587, there was no significant stimulation of GTPγS35 binding (Fig 1E) or cell migration (Fig 1F) under the same assay conditions. Therefore, it appeared that LY2624587 had no apparent agonist activity in the concentrations tested.

### LY2624587 induces receptor internalization and down-regulation of receptor density on the cell surface

**Fig 2:**
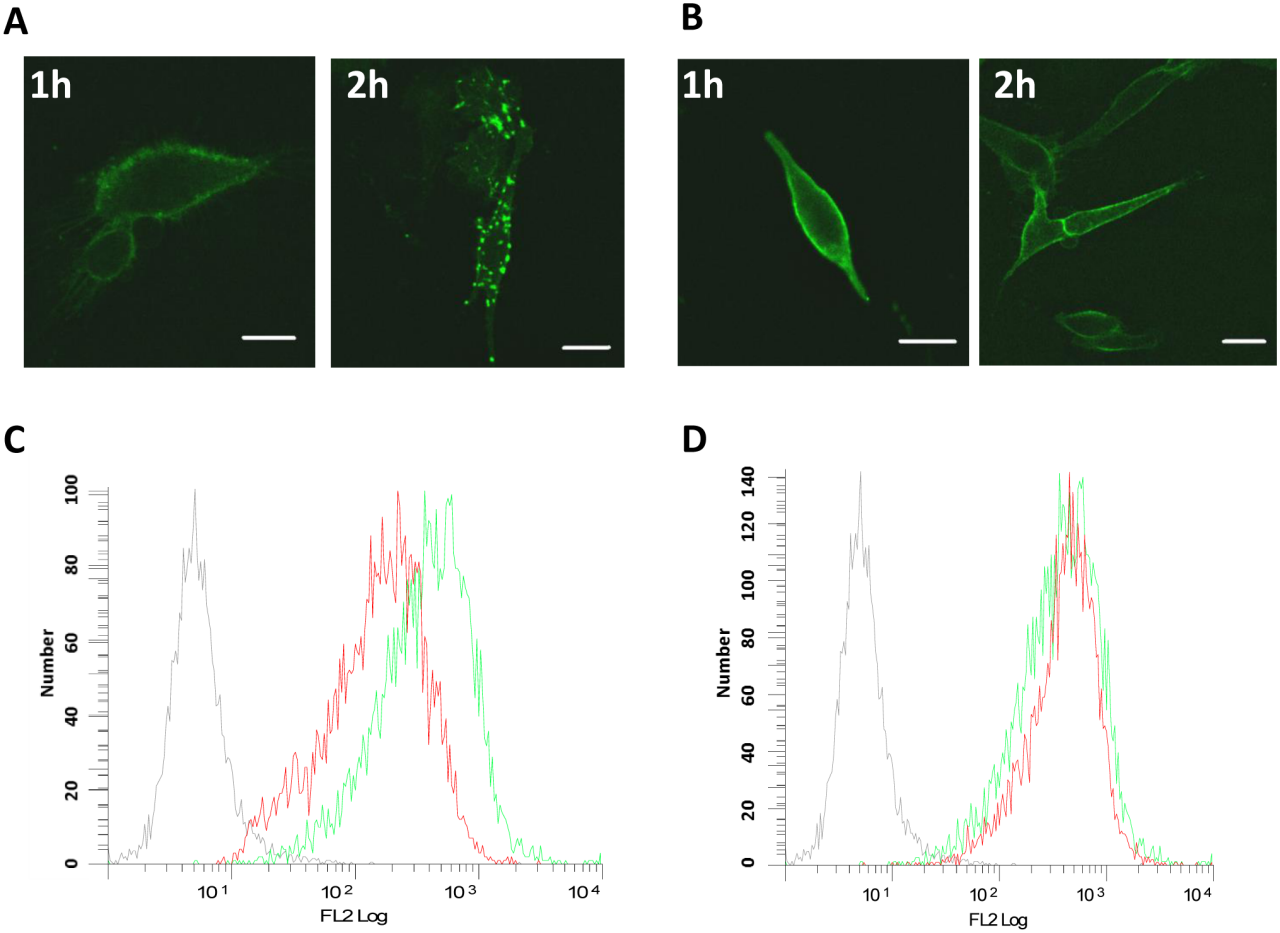
LY2624587 induces CXCR4 receptor internalization and down-regulation of receptor density on the cell surface. LY2624587 was labeled with Alexa 488. The labeled antibody was then used to treat MDA-MB-435/CXCR4 stably transfected cells for 1 or 2 hours at 37°C. After these treatments, the cells were examined under a fluorescent microscope for localization of receptor. In one condition, cells were incubated with labeled LY2624587 for 1 or 2 hours first, then fixed with 2% formaldehyde for 10 min (A). In another condition, the cells was fixed with 2% formaldehyde for 10 min first, then incubated with Alexa 488-labeled LY2624587 for 1 or 2 hours (B). The scale bar is 10 nm. C. LY2624587 induces CXCR4 receptor down-regulation of Namalwa cells. D. Peptide antagonist LY2510924 does not induce CXCR4 receptor down-regulation. NHL Namalwa cells were treated by LY2624587 (C) or LY2510924 (D) for 4 days, and analyzed by flow cytometry with a PE-conjugated anti-CXCR4 antibody that is not competing with LY2624587 or LY2510924 for CXCR4 binding. Grey color was isotype IgG control, green color was no drug treatment; and red color was treated by LY2624587 (C) or LY2510924 (D).

To demonstrate if LY2624587 induced receptor-mediated internalization, the antibody was labeled with Alexa 488. The labeled antibody was then used to treat MDA-MB-435/CXCR4 stably transfected cells, which are adherent cells more suitable for confocal microscopic study. As shown in Fig 2, when cells were treated with 4 μg/mL LY2624587 for 1 or 2 hours, a time-dependent receptor internalization was observed with confocal imaging (Fig 2A). However, if cells were first fixed with 2% formaldehyde in PBS, then treated with LY2624587, there was no significant internalization (Fig 2B). In a separate study, in order to determine LY2624587 could reduce receptor density on the cell surface, we treated Namalwa cells for 4 days with 5μg/ml LY2624587. As illustrated in Fig 2C, flow cytometry analysis showed that LY2624587 indeed significantly reduced CXCR4 receptor density on the surface of Namalwa cells. In contrast, LY2510924, a cyclic peptide CXCR4 antagonist (18), had no effect on cell surface receptor density (Fig 2D).

### LY2624587 inhibits SDF-1 and CXCR4-mediated ERK and AKT phosphorylation in hematologic cells

**Fig 3:**
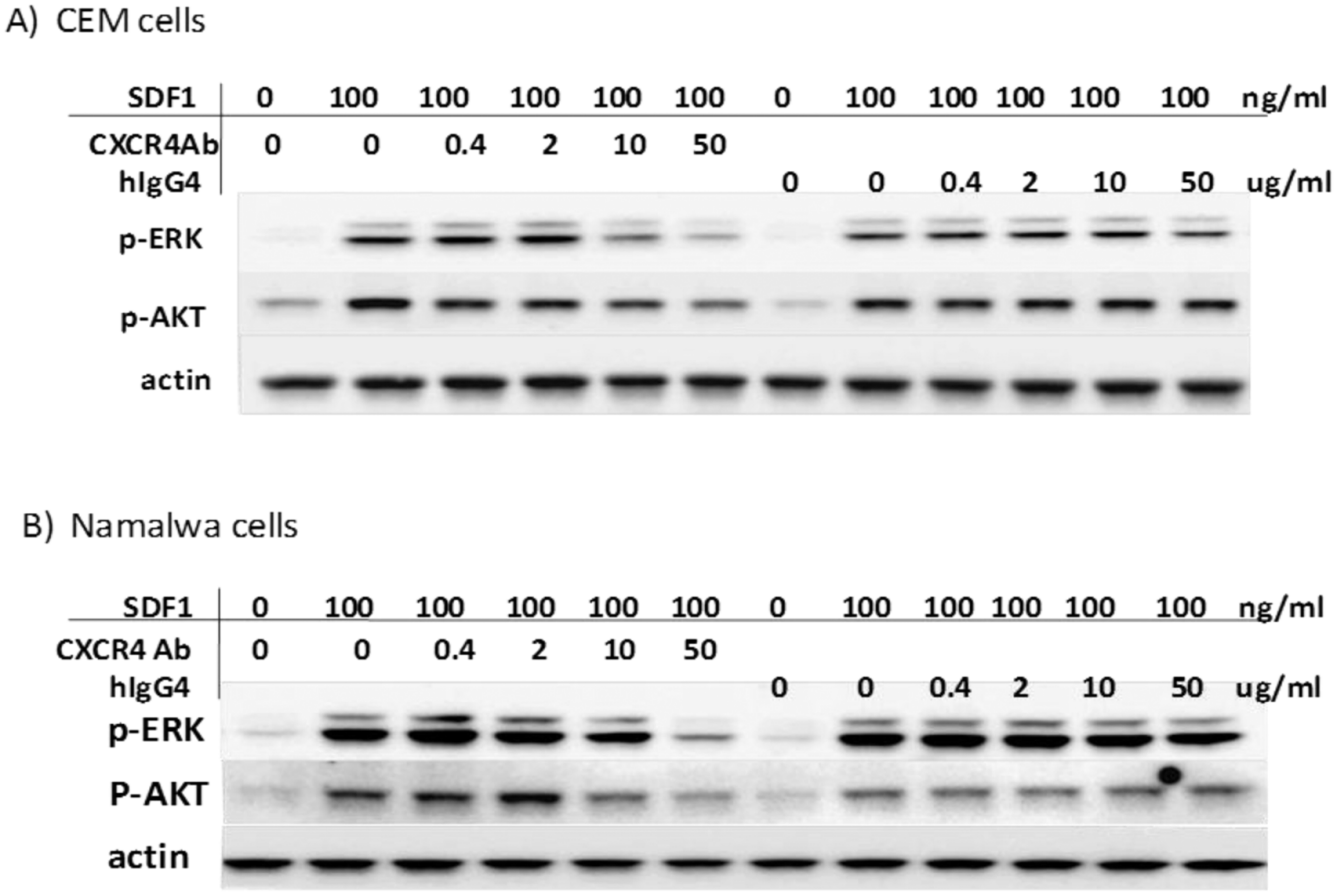
LY2624587 inhibits SDF-1 and CXCR4-mediated cell signaling in tumor cells. Human leukemia CCRF-CEM (A) and NHL Namalwa cells (B)were treated with 100 ng/ml SDF-1 for 10 min in the presence of different concentrations of LY2624587 (CXCR4AB) or control isotype IgG (hIgG4). Western blot analysis of phospho-ERK, phospho-AKT, total ERK or actin was conducted as described under the “Materials and Methods.”

SDF-1/CXCR4 interaction activates a variety of signal transduction pathways, including AKT and ERK. We previously demonstrated that SDF-1 stimulated AKT and ERK phosphorylation in HeLa and Namalwa cells [17, 18]. We characterized the expression of CXCR4 in leukemia (CCRF-CEM) and lymphoma (Namalwa) cells by flow cytometry, and both cell lines expressed high levels of CXCR4 (Fig 2C). Treatment of these cells with SDF-1 at 100 ng/mL significantly stimulated the phosphorylation of ERK and AKT. However, LY2624587 inhibited SDF-1-stimulated ERK and AKT phosphorylation in a concentration dependent manner, while control human IgG4 isotype had no effect under the same conditions (Fig 3). These results suggested that LY2624587 inhibited CXCR4/SDF-1-regulated cell signaling in tumor cells expressing CXCR4.

### LY2624587 induces apoptosis of hematologic malignant cells *in vitro*

**Fig 4:**
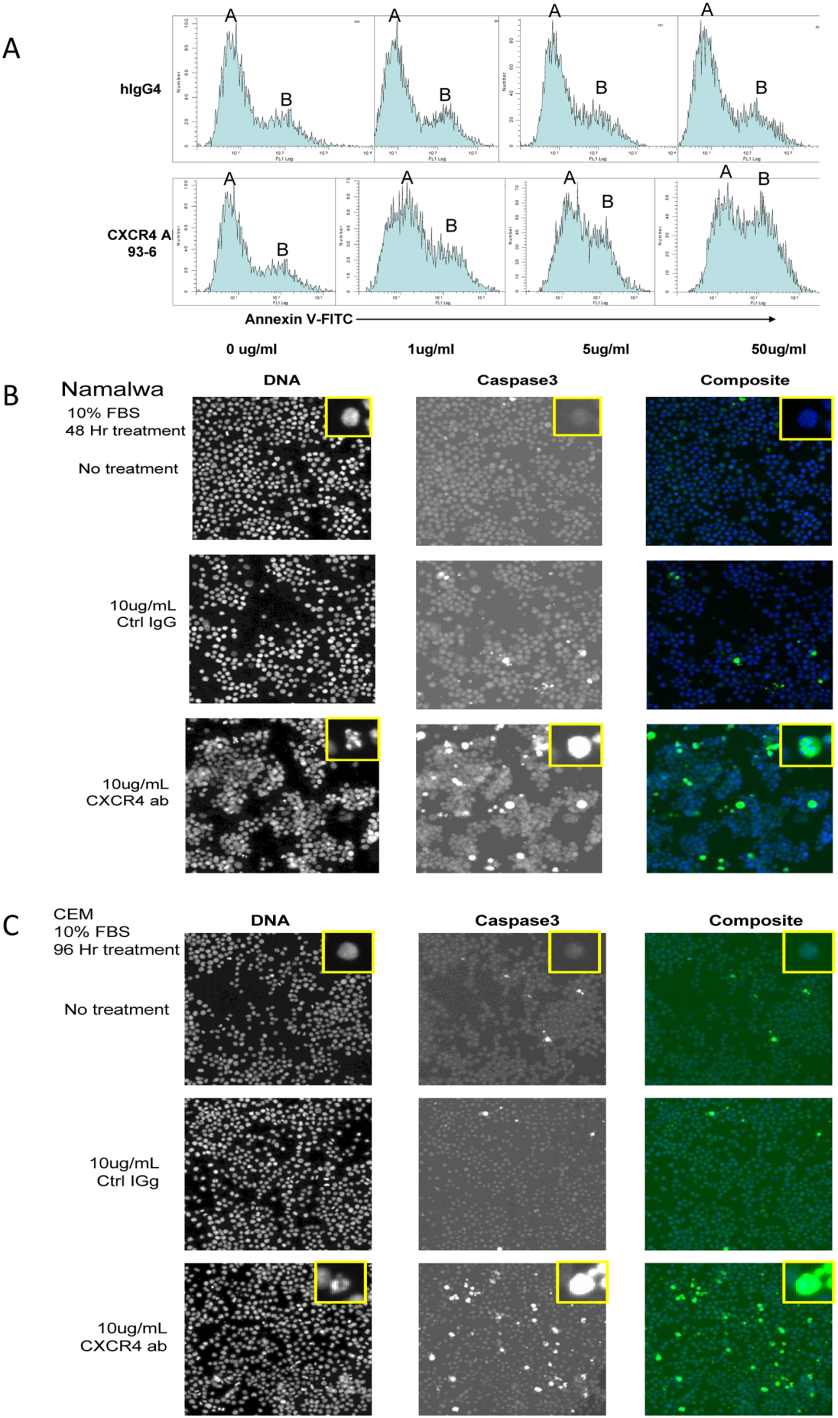
LY2624587 induces apoptosis of hematologic tumor cells *in vitro*. A. Annexin V analysis by flow cytometry in NHL Namalwa cells. Namalwa cells were treated with isotype IgG (hIgG4) or LY2624587 (CXCR4 AB 93–6) for 48 h, then subjected to flow cytometry analysis utilizing FITC-conjugated annexin V. B. DNA fragmentation and cleaved caspase 3 analysis by cellomics analysis in NHL Namalwa cells. The cells were treated for 48 h in the growth medium with 10 μg/ml control IgG or LY2624587 (CXCR4 ab). C. DNA fragmentation and cleaved caspase 3 analysis by cellomics analysis in leukemia CCRF-CEM cells. The cells were treated for 96 h in growth medium with 10 μg/ml control IgG or LY2624587 (CXCR4 ab). The inserts are amplified single cells to show DNA fragmentation or caspase 3 staining.

The CXCR4 and SDF-1 axis is important for tumor cell proliferation and survival. We were interested in assessing if LY2624587 could induce tumor cell apoptosis in vitro. As illustrated in Fig 4A by flow cytometry analysis of annexin V in Namalwa cells that express high levels of CXCR4, LY2624587 induced a concentration-dependent increase in annexin V (peak B), while isotype IgG had no significant effect on the annexin V peak. In contrast, in ARH-77, a hematologic malignant cell line that expresses minimal CXCR4, treatment of LY2624587 had no effect on apoptosis based on annexin V analysis by flow cytometry (S1 Fig). To further confirm LY2624587-induced apoptosis in hematologic malignant cells expressing high levels of CXCR4, we analyzed the changes of the cleaved caspase 3 in Namalwa and CCRF-CEM cells by high content imaging. As demonstrated in Fig 4B, when Namalwa cells were incubated with 10 μg/mL of LY2624587 for 48 hours in the growth medium containing 10% FBS, a significant increase in caspase 3 activity was observed when compared with 10 μg/mL control IgG treatment. At the same time, significant nuclear fragmentation was also observed by LY2624587 treatment. Similarly, when CCRF-CEM cells were incubated with 10 μg/mL LY2624587 for 96 hours, a significant increase in cleaved caspase 3 activity and nuclear fragmentation were also observed (Fig 4C). Since caspase activation and nuclear fragmentation are hallmarks of apoptosis, these results confirmed that LY2624587 indeed induced apoptosis of hematological tumor cells expressing high levels of CXCR4.

### LY2624587 inhibits tumor growth in a solid tumor xenograft model and enhances survival in a disseminated liquid model developed with NHL Namalwa cells

**Fig 5:**
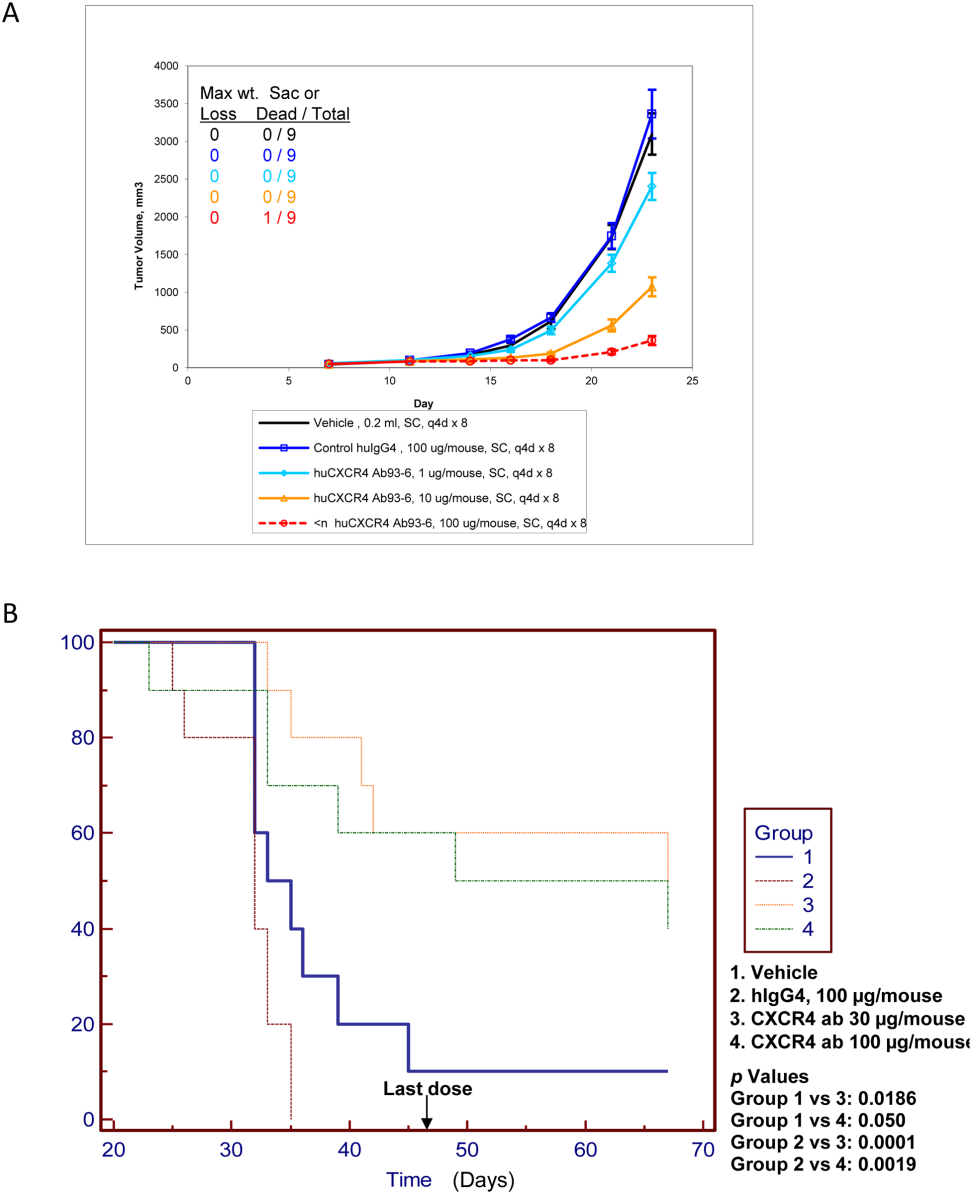
Anti-tumor growth activities of LY2624587 in NHL Namalwa xenograft models developed with SCID mice. A. NHL Namalwa cells were subcutaneously implanted in the rear flank side and grown as solid tumors. B. NHL Namalwa cells were intravenously injected into SCID mice via tail vein, and grown as disseminated lymphomas as described under the “Materials and Methods.”

In order to evaluate in vivo efficacy of LY2624587 in a cancer disease model, we initially established a tumor xenograft model with human NHL Namalwa cells as described [7]. These cells were characterized for CXCR4 expression and function by multiple approaches including CXCR4 expression (Fig 2C and 2D) and CXCR4-dependent ERK and AKT activation (Fig 3B). To test in vivo efficacy of LY2624587 in this model, we treated the animals subcutaneously with 0.04, 0.4 and 4 mg/kg (equivalent to 1, 10 or 100 μg/mouse) of LY2624587 once every 4 days. As demonstrated in Fig 5A, a dose-dependent tumor growth inhibition was observed among compound-treated groups, no significant body weight change was observed in any dose group, and one animal was found dead in 4 mg/kg group in the early time point for unknown reason. Statistical analysis using ANOVA with Dunnett’s comparisons to vehicle or IgG control revealed that all the treatment groups showed statistically significant tumor growth reduction.

To further evaluate in vivo anti-tumor activity of LY2624587, we developed a disseminated liquid model by tail vein injection of NHL Namalwa cells into SCID mice. As shown in Fig 5B, the mice injected with Namalwa tumor cells generally died within 5 to 6 weeks as demonstrated in groups 1 and 2. In vehicle control group, the average survival time was 39 days, and in isotype IgG control group, the average survival time was 30 days. However, treatment of these mice with LY2624587 at 1.2 mg/kg (30 μg/mouse, group 3) or 4 mg/kg (100 μg/mouse, group 4) showed statistically significant survival benefit when compared with isotype IgG control groups in this hematological lymphoma model, and the *p* values are 0.0001 and 0.0019, respectively. In 1.2 and 4 mg/kg treatment groups, the average survival time was 55 and 49.5 days, respectively. It appeared that treatment of 1.2 and 4 mg/kg of LY2624587 showed similar efficacy in this disseminated liquid model although 4 mg/kg treatment demonstrated better efficacy in solid tumor model (Fig 5A). The discrepancy of these anti-tumor activities in these solid and liquid tumor models may link to the local drug concentrations in the tumor microenvironment.

### LY2624587 inhibits tumor growth and induces apoptosis in human leukemia CCRF-CEM xenograft model

**Fig 6:**
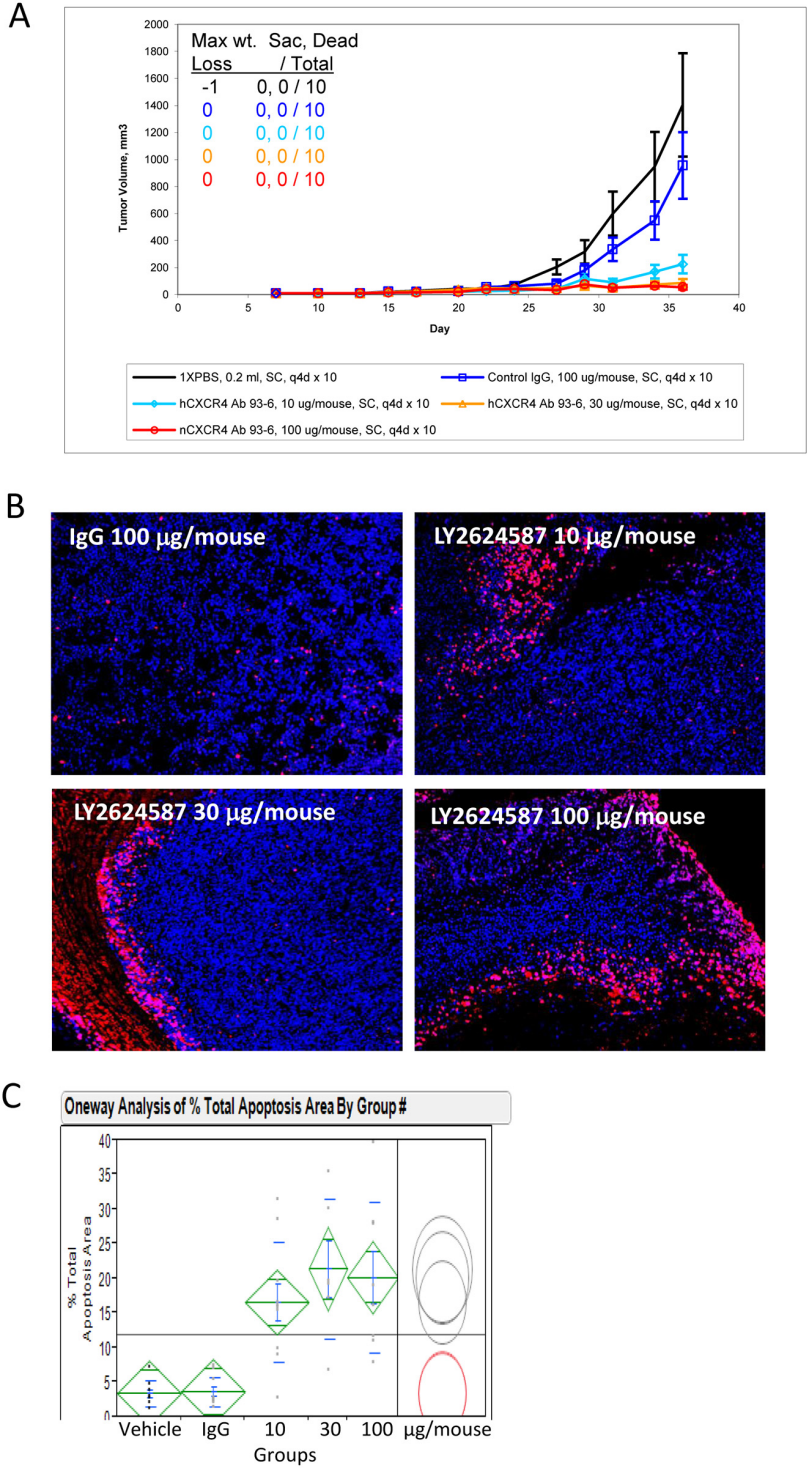
LY2624587 inhibits tumor growth and induces apoptosis of human leukemia cells in CCRF-CEM exnograft model. A. LY2624587 inhibits tumor growth in leukemia CCRF-CEM model. Cells were subcutaneously implanted in the rear flank side and grown as solid tumors. B. LY2624587 induces apoptosis of CCRF-CEM cells *in vivo*. Representative images of multiplexed high content imaging in different treatment groups as indicated, blue, Hoechst staining; red, apoptosis Tunel staining. (C) Quantification and statistical analysis of apoptosis area in different treatment groups as described (27).

CCRF-CEM cells were characterized for CXCR4 expression and function by multiple assays including SDF-1 binding (Fig 1A) and CXCR4-dependent ERK and AKT activation (Fig 3A). 5x10^6^ CCRF-CEM cells were implanted subcutaneously into the rear flank of SCID mice yielding solid tumors. As demonstrated in Fig 6A, treatment of these tumor xenografts with LY2624587 at 0.4, 1.2 or 4 mg/kg led to a dose dependent and statistically significant tumor growth inhibition compared to vehicle and isotype IgG controls. To elucidate the molecular mechanism of anti-tumor activity by LY2624587, we conducted an immuno-histochemistry and high content imaging analysis of the tumor samples [27]. As demonstrated in Fig 6B and S2 Fig, when CCRF-CEM xenograft tissues were sliced and stained with an in situ cell death detection kit for TUNEL staining, a significant increase in TUNEL staining was observed among antibody treatment groups compared with vehicle and isotype IgG controls. Further quantification showed that all antibody treatment groups had statistically higher percentage of total apoptosis area (Fig 6C). For quantification of apoptosis area, multiple 4 micron slides from a whole tumor were scanned and quantified as described [27]. This observation indicated that the CXCR4 antibody LY2624587 indeed induced tumor cell apoptosis in vivo. The results were consistent with the in vitro observation that CXCR4 antibody LY2624587 induced apoptosis in multiple tumor cells including CCRF-CEM cells (Fig 4).

### LY2624587 is capable of binding to human primary CLL cells that express high levels of CXCR4

**Fig 7:**
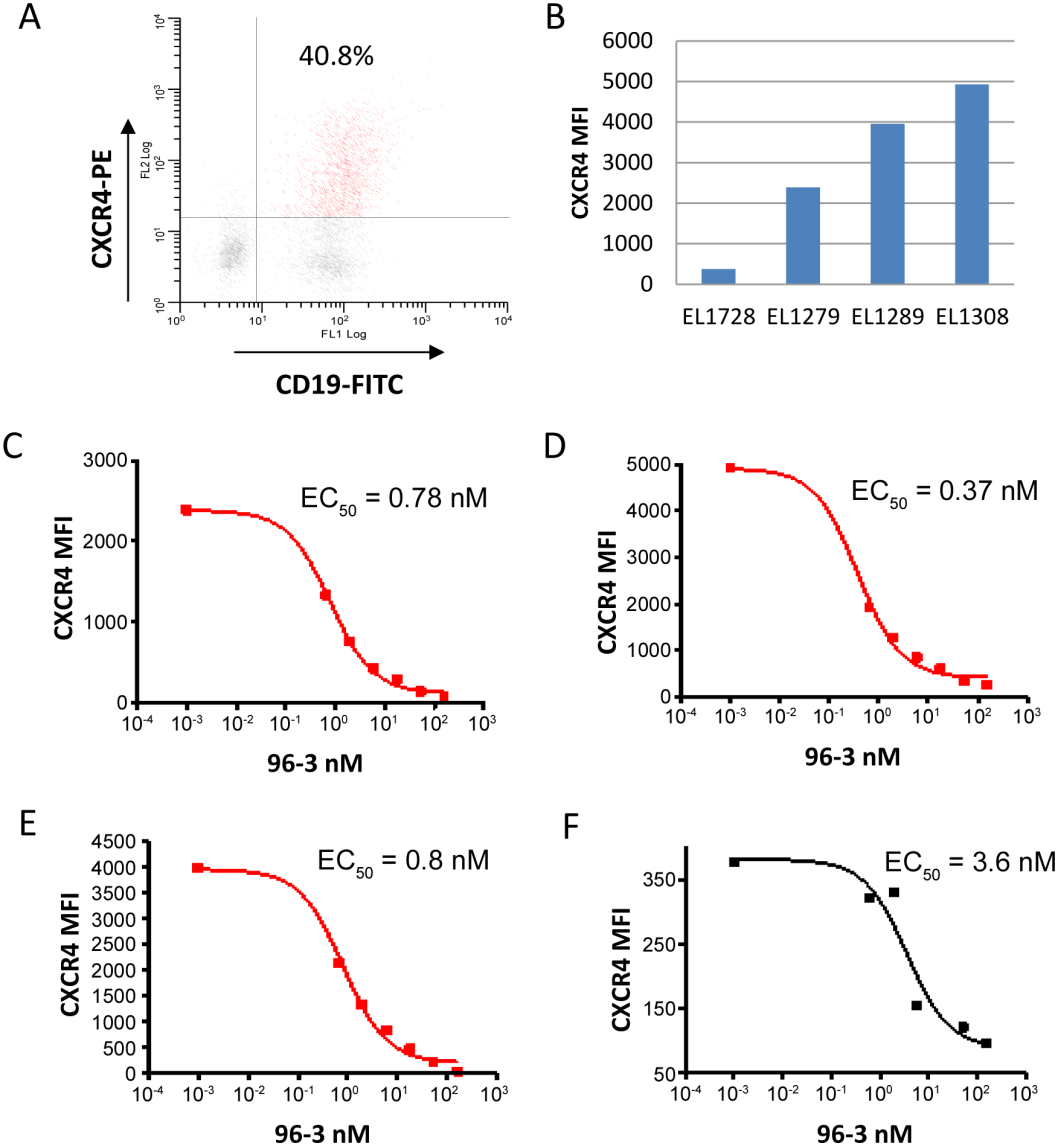
CXCR4 expression in human primary chronic lymphocytic leukemia (CLL) cells and LY2624587 binds to the CXCR4 of CD19+ cells of CCL patients. The blood samples of 4 CLL patients, EL-1279, EL-1308, EL-1289 and EL-1278 were freshly collected from Methodist Hospital, Indiana University at Indianapolis. 20 ml of fresh blood sample from each patient was collected and subjected to flow cytometry analysis with a PE-conjugated CXCR4 antibody and a FITC-conjugated CD19 antibody. A. CXCR4 surface expression in CD19+ cells from a CLL patient. B. CXCR4 intensities in tumor cells of four CCL patients. Three patients express high levels of CXCR4, and one patient has relatively low CXCR4 expression. C-F. LY2624587 (93–6) binds to the CXCR4 of CD19+ cells from CLL patient of EL-1279 (B), EL-1308 (C), EL-1289 (D), and EL-1278 (E). Competitive binding of LY2624587 to CXCR4 was determined using 16 nM of a PE-conjugated CXCR4 antibody from R&D Systems as a tracer. This tracer CXCR4 antibody binds to the same CXCR4 epitope of LY2624587.

To evaluate if tumor cells from CLL patients expressed CXCR4, we identified a commercially available and PE-conjugated CXCR4 antibody (R&D Systems, FAB173P) that competes with LY2624587 for CXCR4 binding. Following patient consent, four individual patient samples were freshly collected from CLL patients from Methodist Hospital of Indiana University. As demonstrated by flow cytometry analysis, CD19+ cells from CLL patients generally displayed a high level expression of CXCR4 (Fig 7A). Based on mean fluorescence intensity (MFI), three out of 4 patient samples, EL-1279, EL-1308 and EL-1278, had high levels of CXCR4 expression, and one patient sample, EL-1278 had a relatively low level of CXCR4 (Fig 7B). Importantly, CXCR4 antibody LY2624587 was able to bind to CXCR4 of CD19+ cells from all 4 patients by competing with PE-conjugated tracer antibody in a dose dependent manner (Fig 7C–7F). In patients with high level of CXCR4, the binding EC50 values were 0.37 to 0.8 nM, similar to what was determined in vitro (Fig 1). In a patient with relatively low CXCR4 expression, the binding EC50 was 3.6 nM. These results suggest that LY2624587 is able to bind the CXCR4 expressed on the primary tumor cells and potentially block CXCR4 function.

## Discussion

The SDF-1 and CXCR4 axis plays an important role in many human diseases including HIV-1 infection, inflammatory diseases, and tumorigenesis. In spite of significant efforts in developing CXCR4 inhibitors for therapeutic use, there is still lack of ideal agents with desirable potency and pharmacokinetic profile suitable for chronic dosing in clinical setting. In recent years, many agents targeting SDF-1 and CXCR4 axis have been developed for clinical use [18–20], and one drug, AMD3100 (plerixafor or Mozobil) was approved for mobilization of hematopoietic stem cells in multiple myeloma and non-Hodgkin lymphoma patients [28, 29]. AMD3100 is a metal-chelating bicyclam, not orally bioavailable, and causes significant safety concerns in chronic dose schedule likely due to compound-associated toxicity [22]. Therefore, this compound can only be administered for short duration treatment in the clinic. LY2624587 described in this study is a fully humanized monoclonal antibody with a human IgG4 backbone and additional mutations in the constant region to eliminate or further reduce antibody effector function. This antibody and BMS-936564/MDX-1338 [25] are among the first in class monoclonal antibodies against CXCR4. Due to the superior pharmacokinetic profile and exquisite selectivity of monoclonal antibody, LY2624587 provides an alternative approach for evaluation of CXCR4 inhibition in treatment of cancer or other diseases. After the completion of phase 1 studies, LY2624587 is currently on hold for additional clinic studies.

Overexpression of the CXCR4 receptor is a hallmark of many hematological malignancies including acute myeloid leukemia (AML), chronic lymphocytic leukemia (CLL), and non-Hodgkin lymphoma (NHL), and generally correlated with more invasiveness, high risk of the disease and poor prognosis [7, 11, 12, 25, 30–33]. CXCR4 and SDF-1 axis is important for hematological tumor cell survival, migration and interaction with their protective microenvironment. In this study, we have demonstrated that human primary CLL cells generally express high level of CXCR4, and LY2624587 is able to block SDF-1 and CXCR4 mediated cell signaling and induce apoptosis of NHL and lymphoblastic leukemia cells in vitro. It has single agent anti-tumor growth activities in xenograft models of hematological malignancies. Therefore, LY2624587 has a potential for treatment of hematological malignancies as a single agent or in combination with other agents in clinic. The results are consistent with a previous report in which a CXCR4 antibody showed antitumor activity in AML and NHL preclinical models [25]. Additionally, a CXCR4 peptide antagonist LY2510924 and BKT140 also showed anti-tumor activities in lymphoma xenograft models [18, 33, 34]. In a previous study, CXCR4 inhibition by AMD3100 or TN140 selectively eliminated CXCR4-expressing human AML cells while no effect was observed for CXCR4 negative or low AML cells to confirm the role of CXCR4 expression in this disease [35]. Our study also demonstrated that LY2624587-induced apoptosis occurred only in hematologic tumor cells with high level CXCR4 expression. More importantly, LY2624587 may be combined with other standard of care for treatment of hematological malignancies. Indeed, it was demonstrated that CXCR4 inhibition was efficacious in AML or multiple myeloma models by enhancing the sensitivity of tumor cells to chemotherapy or other targeted therapy [33, 34, 36, 37]. A clinical trial in combining CXCR4 inhibitor AMD3100 and standard of care for AML was conducted [11].

SDF-1 and CXCR4 interaction activates multiple signaling pathways, including PI3K/AKT, PLC-γ/protein kinase c, and MAPK cascade [16, 17, 38]. Functionally, CXCR4 plays a role in many phases of tumor biology, including tumor growth, survival, invasion, and metastasis. The molecular mechanism of anti-tumor activities by SDF-1 and CXCR4 inhibition could be complex in different models. In this study, we showed that LY2624587 caused apoptosis of hematological tumor cells in vitro and in vivo. In vitro, CXCR4 inhibition by LY2624587 leads to the cell apoptosis in tumor cells expressing high levels of CXCR4. This in vitro apoptotic effect was further confirmed in leukemia CEM model in vivo (Fig 6B). In fact, CXCR4 inhibition by LY2510924, a selective and small peptide CXCR4 antagonist without any effector function also induced apoptosis in vivo in the same leukemia CEM model (unpublished observations). CXCR4 monoclonal antibody BMS-936564/MDX-1388 was previously demonstrated to induce apoptosis of hematologic tumor cells [25]. The difference between LY2624587 and BMS-936564 is that LY2624587 is a fully humanized monoclonal antibody while BMS-936564 is a human antibody generated in Medarex KM transgenic mice [25]. More importantly, LY2624587 has a much higher binding affinity to CXCR4 and more potent cellular activities. For example, in the same ligand binding assay developed with CCRF-CEM cells, LY2624587 has a binding affinity of 0.26 nM, and BMS-936564 showed a binding affinity of 4.3 nM [25], a 16 fold difference. The high binding affinity of LY2624587 was further confirmed in patient CLL cells (Fig 7).

Since LY2624587 has low or nonexistent potential to stimulate effector functions, the in vivo activities observed in this study cannot simply be explained by antibody-dependent cell-mediated cytotoxicity or complement-dependent cytotoxicity. The observation that LY2624587 induced apoptosis in the absence of added ligand could be explained by a combination of the following two reasons. First, these cells may secrete SDF-1 and promote an autocrine effect as described [4], and second, LY2624587- induced receptor internalization and receptor down regulation on the cell surface described in this study may also contribute to the apoptosis. Further studies in investigating the molecular mechanism of LY2624587-induced apoptosis in hematological tumor cells are ongoing. In addition to hematological tumor cells, CXCR4 inhibition has also been reported to induce apoptosis in many other tumor cells. In oral squamous cell carcinoma, RNAi-mediated CXCR4 silencing caused tumor cell apoptosis in vitro and in vivo [39]. In pituitary tumor cells, CXCR4 inhibition by a peptide antagonist induced apoptosis through activation of the caspase-3 pathway [40]. Depending on CXCR4 expression and specific genetic and microenvironment background, SDF-1 and CXCR4 axis appears to be an important survival mechanism in tumor cells.

Altogether, we have described a fully humanized CXCR4 monoclonal antibody that was advanced into the clinic for treatment of cancer. This antibody induces apoptosis of hematological tumor cells in vitro and in vivo, and has the therapeutic potential as either a single agent or in combination for the treatment of hematological tumors, including but not limited to leukemia and lymphomas.

## Supporting Information

S1 Fig Annexin V analysis by flow cytometry in hematologic tumor ARH-77 cells expressing low levels of CXCR4. ARH-77 cells were treated with isotype IgG4 (C) or LY2624587 (CXCR4 Ab) at the indicated concentrations (D-F) for 48 h, then subjected to flow cytometry analysis utilizing FITC-conjugated annexin V and propidium iodide. 100nM Staurosporine 24hr treatment was used as a positive control (B). LY2624587 treatment did not induce significant apoptosis of ARH-77 cells compared with isotype IgG4 control.
(PDF)

S2 Fig LY2624587 induces apoptosis of CCRF-CEM cells in xenograft tumors. Representative images of multiplexed high content imaging in different treatment groups shown in Fig 6A were presented. Blue, Hoechst staining; red, apoptosis Tunel staining.
(PDF)
