# Supplementary figures and images for: Inhibition of CXCR4 by LY2624587, a Fully Humanized Anti-CXCR4 Antibody Induces Apoptosis of Hematologic Malignancies
Source: PLoS One. 2016 Mar 8;11(3):e0150585. doi: 10.1371/journal.pone.0150585 (PMC4782998; doi:10.1371/journal.pone.0150585)

**A**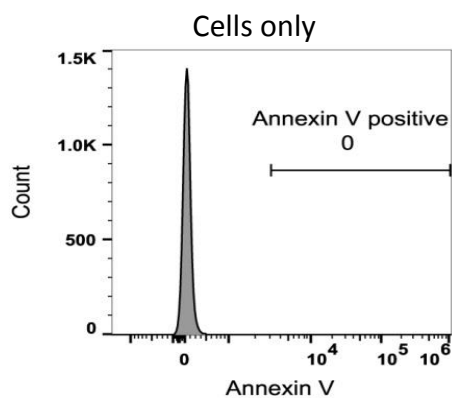**B**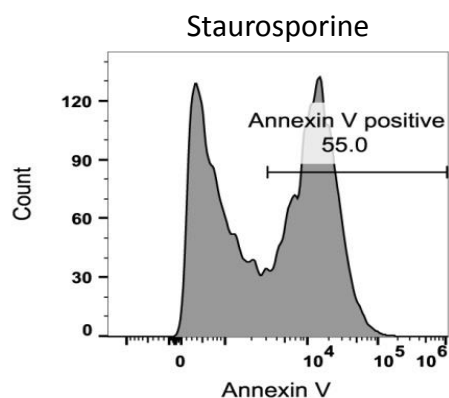**C**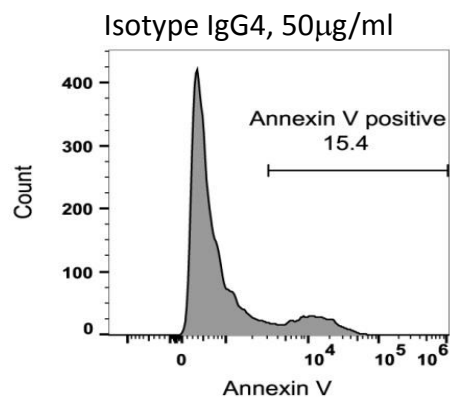**D**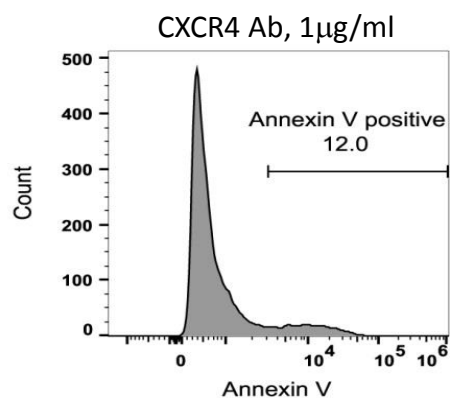**E**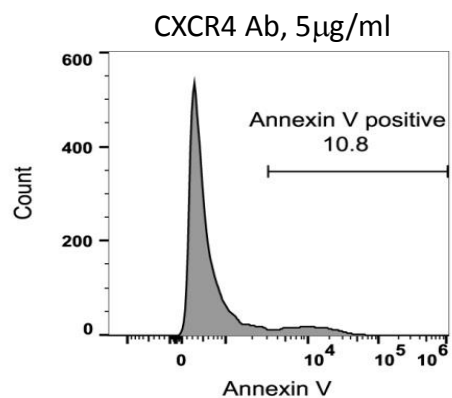**F**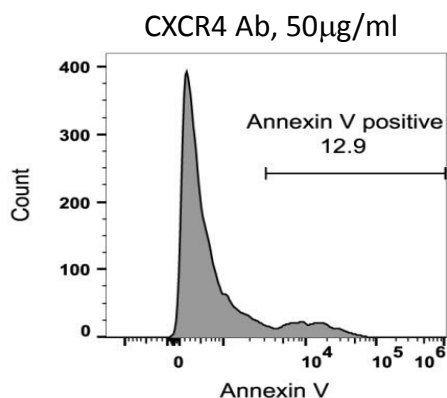

Supplement: S1 Fig — ARH-77 cells were treated with isotype IgG4 (C) or LY2624587 (CXCR4 Ab) at the indicated concentrations (D-F) for 48 h, then subjected to flow cytometry analysis utilizing FITC-conjugated annexin V and propidium iodide. 100nM Staurosporine 24hr treatment was used as a positive control (B). LY2624587 treatment did not induce significant apoptosis of ARH-77 cells compared with isotype IgG4 control. (PDF) [file pone.0150585.s001.pdf]

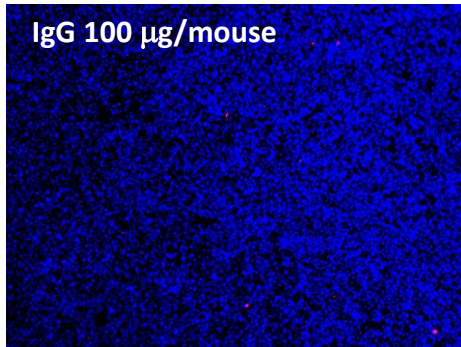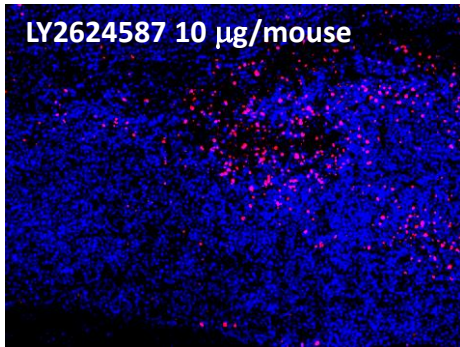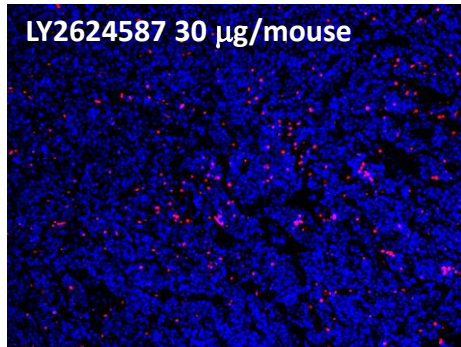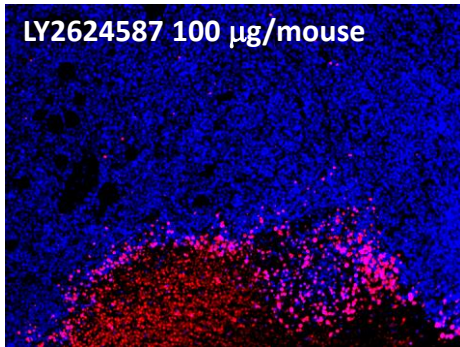

Supplement: S2 Fig — Representative images of multiplexed high content imaging in different treatment groups shown in Fig 6A were presented. Blue, Hoechst staining; red, apoptosis Tunel staining. (PDF) [file pone.0150585.s002.pdf]
